# Characterizing the childhood roots of adult sense of mastery across 22 countries in the global flourishing study

**DOI:** 10.1038/s41598-025-03045-0

**Published:** 2025-05-27

**Authors:** Eric S. Kim, Matt Bradshaw, Ying Chen, William J. Chopik, Sakurako S. Okuzono, Renae Wilkinson, R. Noah Padgett, Margie E. Lachman, Byron R. Johnson, Tyler J. VanderWeele

**Affiliations:** 1 Department of Psychology, University of British Columbia, Vancouver, Canada; 2 Human Flourishing Program, Institute for Quantitative Social Science, Harvard University, Cambridge, MA USA; 3 Lee Kum Sheung Center for Health and Happiness, Harvard T.H. Chan School of Public Health, Boston, MA USA; 4 Institute for Studies of Religion, Baylor University, Waco, TX USA; 5 Department of Epidemiology, Harvard T.H. Chan School of Public Health, Boston, MA USA; 6 Department of Psychology, Michigan State University, East Lansing, MI USA; 7 Department of Social & Behavioral Sciences, Harvard T.H. Chan School of Public Health, Boston, MA USA; 8 Department of Psychology, Brandeis University, Waltham, MA USA; 9 School of Public Policy, Pepperdine University, Malibu, CA USA; 10 Department of Biostatistics, Harvard T.H. Chan School of Public Health, Boston, MA USA

**Keywords:** Human behaviour, Epidemiology

## Abstract

How might we cultivate a life imbued with a sense of mastery? An expanding body of research demonstrates that a heightened sense of mastery improves health and well-being outcomes. Despite this, it remains unclear which childhood factors foster increased mastery in adulthood. Further, existing studies have examined this question only within single countries. We analyzed nationally representative data from 22 countries in the Global Flourishing Study (*N* = 202,898) and evaluated if 11 aspects of a child’s upbringing predict mastery in adulthood, and also whether these associations vary by country. Some childhood factors were associated with increased mastery in adulthood, including good health, good relationships with mothers and fathers, economic stability, and regular religious service attendance. Childhood factors associated with decreased mastery in adulthood included abuse, feeling like an outsider in one’s family, poor health, economic hardship, and being female. However, there was little evidence that parent marital status or immigration status in childhood were associated with mastery in adulthood. Our meta-analysis also revealed substantial heterogeneity in childhood pathways to adult mastery across 22 countries. With further research, these findings could inform the development of globally adaptable, yet locally nuanced, programs and policies designed to foster a mastery across the globe.

## Introduction

How might we cultivate a life infused with mastery? A small but emerging body of research is beginning to shed light on this question, but to our knowledge, most existing studies have examined this question only within single and mostly “WEIRD” societies (Western, Educated, Industrialized, Rich, and Democratic)^1^. Cross-national research offers a unique opportunity to deepen our understanding of how contrasting sociopolitical and economic environments shape people’s sense of mastery (referred to as either “mastery” or “sense of mastery” throughout this paper). Assessing how various childhood predictors influence adult mastery across national climates and contexts can help us understand which factors consistently influence mastery globally, and which are amplified (or dampened) by specific cultural contexts. However, to date, these questions have arguably been underexplored. Bridging these knowledge gaps could enhance our understanding of the developmental roots of mastery and also highlight potential areas for early intervention.

Sense of mastery—the perception that one has the ability to influence their environment and elicit desired outcomes—^2,3^ is important in its own right, but also because it shapes trajectories of psychological-, social-, behavioral-, and physical-well-being. Several hypothesized mechanisms help explain the potentially salubrious effects of mastery, including: (1) better health behaviors (e.g., increased: physical activity, decreased: sleep problems)^4,5^, (2) enhanced psychological well-being (e.g., purpose in life, positive affect, optimism)^4,6^, and decreased psychological distress (e.g., negative affect, depressive symptoms, and anxiety)^4,6^. In line with this emerging evidence is work showing that mastery is associated with reduced risk of chronic conditions (e.g., cognitive impairment, stroke, cardiovascular disease)^4,5,7,8^ and mortality^4,9^. Further, many national and international strategies to promote population health include increasing mastery as a central recommendation for action^10,11^.

Sense of mastery is influenced by genetics, social-structural factors, and changing life circumstances^12,13^. Further, it can be contextualized within Albert Bandura’s social cognitive theory^3^. Specifically, a person’s sense of mastery is influenced by (1) firsthand experiences of mastery, (2) feedback from interactions with others, and (3) affective and emotional reactions. The impact of these three factors can vary substantially across different cultural contexts. For instance, cultures that emphasize individual achievement might highlight the role of personal performance experiences, whereas cultures valuing collective success and community support may strengthen the influence of social feedback on an individual’s sense of mastery. This perspective underscores the complexity of mastery as not only stemming from within an individual but also as being substantially shaped by external environments, social interactions, and culture.

Sense of mastery is potentially modifiable through interventions (e.g., instilling beliefs that mastery is malleable, a cognitive behavioral therapy that focuses on restructuring maladaptive thoughts around mastery beliefs, increasing autonomy [e.g., how and when leisure-activities or work tasks are performed])^2,14,15^. However, not many interventions have been developed, and one obstacle hindering intervention development is the identification of antecedents that predict mastery. Previous studies have focused on adulthood, where higher mastery is predicted by potentially modifiable factors assessed during this life stage, including social factors (e.g., higher: social participation, volunteering, social network contact, perceived support from network members; lower: loneliness)^9,16–22^, psychological factors (e.g., decreased: depression and negative affect)^19,22,23^, cognitive abilities^24^, and physical health factors (decreased: physical functioning, chronic illnesses)^16,22,25^.

These prior studies have made critical contributions to the research literature but remain somewhat limited for at least five reasons. First, research directly examining childhood predictors of adult mastery is sparse. And targeting health assets, such as mastery, during childhood—a critical developmental phase for acquiring health assets and establishing healthy behaviors and mindsets—is a promising point of intervention that can enhance the trajectory of health and well-being across the life course^26,27^. Second, nearly all existing studies have been confined to examining predictors of mastery within single and mostly “WEIRD” societies^1^. Doing so limits our ability to evaluate how the sociopolitical and economic contexts of different countries might shape sense of mastery. Third, many studies use data from small samples or specific subpopulations, limiting their generalizability to broader populations. Fourth, many studies did not adequately adjust for key potential confounders (e.g., only adjusting for basic demographics). Fifth, most studies only evaluated a limited number of predictors, so that we cannot directly compare effect sizes between different early life exposures, which is helpful when trying to determine intervention targets that might produce the largest effects.

In response to these gaps in the literature, our study aimed to address several of these limitations. Using data from a diverse and international sample of 202,898 people across 22 countries, and using an exposure-wide analytic approach (see Statistical Analysis section), our study sought to answer three central questions: #1) How do 11 aspects of a child’s upbringing predict mastery in adulthood? #2) Do these associations vary by country? #3) Are the observed relationships robust to potential unmeasured confounding variables? This hypothesis-generating, data-driven approach allowed us to identify promising antecedents of mastery, which can then undergo further investigation in future studies.

## Methods

The description of the methods below have been adapted from VanderWeele et al.^28^. Further methodological detail is available elsewhere^29–36^.

### Study population

We used data from the Global Flourishing Study (GFS), which examines the distribution and determinants of well-being across a sample of 202,898 participants from 22 geographically and culturally diverse countries. Wave 1 of GFS collected nationally representative data from the following countries and territories: Argentina, Australia, Brazil, Egypt, Germany, Hong Kong (Special Administrative Region of China), India, Indonesia, Israel, Japan, Kenya, Mexico, Nigeria, the Philippines, Poland, South Africa, Spain, Sweden, Tanzania, Türkiye, United Kingdom, and the United States. These countries were chosen to (1) maximize coverage of the world’s population, (2) ensure geographic, cultural, and religious diversity, and (3) prioritize feasibility and existing data collection infrastructure. Gallup Inc conducted the data collection primarily in 2023, although some regions began in 2022; the timing of assessments varied by country, and more information can be found elsewhere^52^. The precise sampling design to ensure nationally representative samples varied by country and further details are available elsewhere^33^. The data are publicly available through the Center for Open Science (https://www.cos.io/gfs). The translation process followed the TRAPD model (translation, review, adjudication, pretesting, and documentation) for cross-cultural survey research (ccsg.isr.umich.edu/chapters/translation/overview). Translations of all survey questions are available here: https://osf.io/d4qw8. Further details about methodology and survey development are documented in the GFS Questionnaire Development Report^29^, GFS Methodology^33^, GFS Codebook^37^, and GFS Translations documents^38,39^. Ethical approval was granted by the Institutional Review Boards at Baylor University (IRB Reference #: 1841317) and Gallup Inc. (IRB Reference #: 2021-11-02). Gallup is a multi-national corporation and its IRB covers all countries included in the GFS. All participants provided informed consent to Gallup and IRB approval for all data collection activities was obtained by Gallup (10.1007/s10654-024-01167-9). IRB approval for data analysis was granted by Baylor University. All personally identifiable information (PII) was removed from the data used in this study by Gallup, and was not accessible to the authors. This research conformed to the principles of the Helsinki Declaration. No further IRB approval was needed for our secondary analyses.

### Measures

#### Sense of mastery

 Sense of mastery was assessed by asking “How often do you feel very capable in most things you do in life?” Response options included: Always, often, rarely, never. In our main analyses, mastery was dichotomized as 1 (always/often) vs. 0 (rarely/never)—as described in our pre-registered analyses. We initially chose this cutoff because it reflects a conceptual distinction where individuals who consistently feel capable (“Always/Often”) are separated from those who have a pronounced deficit in perceived capability (“Rarely/Never”). However, as a post-hoc sensitivity analysis, mastery was analyzed with a different dichotomization point: 1 (always) vs. 0 (often/rarely/never). The distribution of responses to the mastery question was: “Always” (26.14%, *n* = 53,041), “Often” (51.12%, *n* = 103,728), “Rarely” (18.94%, *n* = 38,433), and “Never” (3.25%, *n* = 6,90). Additionally, 0.45% (*n* = 906) skipped the question, 0.09% (*n* = 178) did not know, and 0.01% (*n* = 22) refused, totaling 0.55% (*n* = 1,06) non-responses.

#### Candidate childhood predictors

 We evaluated 11 candidate predictors that were assessed at baseline. Each predictor is numbered below (#1-#11) and organized into five categories (A-E).

A) Demographic predictors, included: #1) Year of Birth/current age (“What is your age? Please enter your age below.” Year of birth/current age was classified as follows: 1998–2005 (18–24 years), 1993–1998 (25–29 years), 1983–1993 (30–39 years), 1973–1983 (40–49 years), 1963–1973 (50–59 years), 1953–1963 (60–69 years), 1943–1953 (70–79 years), and 1943 or earlier (80 + years), #2) Gender (“What is your gender?” Response options were: Male, Female, or Other), and #3) Immigration status (“Were you born in this country, or not?” Response options were: Yes or No). Race/ethnicity was also assessed in some countries and when available was included as a childhood predictor by categorizing race as a binary indicator of whether an individual was in the most prominent racial/ethnic group within country or not, but since the categories used to assess race/ethnic identity varied by country, we did not meta-analyze this effect.

B) Socioeconomic predictors, included: #4) Subjective financial status of family growing up: (“Which one of these phrases comes closest to your own feelings about your family’s household income when you were growing up, such as when YOU were around 12 years old?” Response options were: Lived comfortably, Got by, Found it difficult, or Found it very difficult).

C) Family dynamics and relationship predictors, included: #5) Parent marital status / Family structure (“Were your parents married to each other when YOU were around 12 years old?” Response options were: Married, Divorced, Never married, One or both had died), #6) Relationship with mother (“Please think about your relationship with your mother when you were growing up. In general, would you say that relationship was…” Response options were: Very good, Somewhat good, Somewhat bad, or Very bad?” Responses were dichotomized to Very/Somewhat good versus Very/Somewhat bad), #7) Relationship with father (An analogous question and answers were used to assess relationship with father). “Does not apply” was treated as a dichotomous control variable for respondents who did not have a mother or father due to death or absence, #8) Felt like an outsider in family growing up (“When you were growing up, did you feel like an outsider in your family?” Response options were: Yes or No), and #9) Abuse (“Were you ever physically or sexually abused when you were growing up?” Response options were: Yes or No).

D) Health and well-being predictors, included: #10) Self-rated health growing up (“In general, how was your health when you were growing up?” Response options were: Excellent, Very good, Good, Fair, or Poor?”).

E) Religious and spiritual predictors, included: #11) Age 12 religious service attendance (“How often did YOU attend religious services or worship at a temple, mosque, shrine, church, or other religious building when YOU were around 12 years old?” Response options were: At least once/week, One-to-three times/month, Less than once/month, or Never). Childhood religious tradition/affiliation had response categories of Christianity, Islam, Hinduism, Buddhism, Judaism, Sikhism, Baha’i, Jainism, Shinto, Taoism, Confucianism, Primal/Animist/Folk religion, Spiritism, African-Derived, some other religion, or no religion/atheist/agnostic; precise response categories varied by country^38^. This latter factor was analyzed as a childhood predictor by using the most prominent religious tradition within country as the reference group or no religion/atheist/agnostic as the reference group if the within country group size was greater than 5% with the within country sample. Due to the varying reference group, this effect was not meta-analyzed.

### Statistical analysis

Descriptive statistics for the observed sample, weighted to be nationally representative within country, were estimated for each childhood demographic category. A weighted modified Poisson regression model with complex survey adjusted standard errors was fit within each country for mastery on all of the aforementioned childhood predictor variables simultaneously. In the primary analyses, we conducted random effects meta-analyses of the regression coefficients^40,41^ along with confidence intervals, the proportion of calibrated effects by threshold (above a RR of 1.10 or below a RR of 0.90), and I^2^ for evidence concerning variation within a given demographic category across countries^42^. We used a random effects approach using the Paule and Mandel estimator to calculate the between-country variance (^2^)^43–45^, and assumed that true effect sizes across countries arise from a normal distribution. Forest plots of estimates are available in the online supplement, and they include tau estimates. Religious affiliation/tradition and race/ethnicity were used within country as control variables, when available, but these coefficients themselves were not included in the meta-analyses because categories/responses varied by country. All meta-analyses were conducted in R (R Core Team, 2024) using the “metafor” package^46^.

Within each country, a Wald-type global test of association of each childhood predictor variable group with outcome was conducted, and a pooled *p*-value^47^ across countries reported evidence for an association within *any* country. Bonferroni corrected *p*-value thresholds are provided based on the number of childhood demographic variables^48,49^. For each childhood predictor, we calculated E-values to evaluate the sensitivity of results to potential unmeasured confounding. An E-value is the minimum strength of the association an unmeasured confounder must have with both the outcome and the predictor, above and beyond all measured covariates, for an unmeasured confounder to explain away an association^50^. As a supplementary analysis, population weighted meta-analyses of the regression coefficients were estimated. All analyses were pre-registered with the Center for Open Science (OSF) prior to data access, with only slight modifications in the regression analyses due to multicollinearity, which were reported in an addendum to our pre-registration https://osf.io/yrn8h. All code to reproduce analyses are openly available in the OSF online repository: https://osf.io/vbype/?view_only=0372838c315d46a995c122f9c637ae5d^31^.

### Missing data

Missing data on all variables was imputed using multivariate imputation by chained equations creating five imputed datasets^51–53^. To account for variation in the assessment of certain variables across countries (e.g., religious affiliation/tradition and race/ethnicity), the imputation process was conducted separately in each country. This within-country imputation approach ensured that the imputation models accurately reflected country-specific contexts and assessment methods. Sampling weights were included in the imputation model to account for missingness to be related to probability of inclusion.

### Accounting for complex sampling design

The GFS used different sampling schemes across countries based on the availability of existing panels and recruitment needs^33^. All analyses accounted for the complex survey design components by including weights, primary sampling units, and strata. Sampling weights for each country were constructed by Gallup to account for selection probability, coverage, and non-response, ensuring that estimates are nationally representative. Full details regarding the weighting methodology, including how the weights were developed and validated, can be found in our companion analytic methods paper^32^. Additional methodological detail, including accounting for the complex sampling design, is provided elsewhere^32^.

### AI use statement

The authors used Claude, an AI language model, to assist with wording refinements and clarity of expression in this manuscript. All ideas, arguments, and substantive content remain the original work of the authors.

## Results

### Descriptive statistics

Table 1 provides the distribution of descriptive statistics. Participant ages spanned the entire adult lifespan (18–80+). The gender distribution was nearly balanced, with 51% female, 49% male, and a small representation from other gender identities (< 1%). Most participants had a very good or good relationship with their mothers (89%) and fathers (80%), and 75% reported their parents were married during their childhood. In terms of childhood financial environment, 35% lived comfortably, while 41% got by, 18% found it difficult, and 6% found it very difficult. The majority of the sample reported no childhood abuse (82%), while 14% reported abuse. 33% reported having excellent health and 31% reported very good health. The vast majority were native-born (94%). Regular attendance at religious services during childhood varied, with 41% attending at least once a week, 23% never attending, and 18% attending less than once a month.

**Table 1:** Nationally-Representative descriptive statistics of the observed sample (*N* = 202,898)^a, B^.

Variable	Proportion	Frequency
Relationship with mother		
Very Good	63%	127,836
Somewhat Good	26%	52,439
Somewhat Bad	5%	11,060
Very Bad	2%	4,642
Not Applicable	3%	5,965
Missing	0%	956
Relationship with Father		
Very Good	53%	107,742
Somewhat Good	27%	55,714
Somewhat Bad	8%	15,807
Very Bad	4%	8,278
Not Applicable	7%	13,985
Missing	1%	1,372
Parent Marital Status		
Married	75%	152,001
Divorced	9%	17,726
Never Married	8%	15,534
One or Both Had Died	4%	7,794
Missing	5%	9,843
Childhood Income		
Lived Comfortably	35%	70,861
Got By	41%	82,905
Found it Difficult	18%	35,852
Found it Very Difficult	6%	12,606
Missing	0%	674
Childhood Abuse		
Yes	14%	29,139
No	82%	167,279
Missing	3%	6,479
Outsider		
Yes	14%	28,732
No	84%	170,577
Not Applicable	1%	2,712
Missing	0%	877
Childhood Health		
Excellent	33%	67,121
Very Good	31%	63,086
Good	23%	47,378
Fair	10%	19,877
Poor	2%	4,906
Missing	0%	530
Immigration Status		
Born in This Country	94%	190,998
Born in Another Country	5%	9,791
Missing	1%	2,110
Childhood Service Attendance		
At Least 1/Week	41%	83,237
1–3/Month	16%	33,308
<1/Month	18%	36,928
Never	23%	47,445
Missing	1%	1,980
Gender		
Male	49%	98,411
Female	51%	103,488
Other	0%	602
Missing	0%	397
Year of Birth		
1998–2005; Age 18–24	13%	27,007
1993–1998; Age 25–29	10%	20,700
1983–1993; Age 30–39	20%	40,256
1973–1983; Age 40–49	17%	34,464
1963–1973; Age 50–59	16%	31,793
1953–1963; Age 60–69	14%	27,763
1943–1953; Age 70–79	8%	16,776
1943 or Earlier; 80 or Older	2%	4,119
Missing	0%	20
Country		
Argentina	3%	6,724
Australia	2%	3,844
Brazil	7%	13,204
Egypt	2%	4,729
Germany	5%	9,506
Hong Kong	1%	3,012
India	6%	12,765
Indonesia	3%	6,992
Israel	2%	3,669
Japan	10%	20,543
Kenya	6%	11,389
Mexico	3%	5,776
Nigeria	3%	6,827
Philippines	3%	5,292
Poland	5%	10,389
South Africa	1%	2,651
Spain	3%	6,290
Sweden	7%	15,068
Tanzania	4%	9,075
Turkey	1%	1,473
United Kingdom	3%	5,368
United States	19%	38,312

^a^Country-specific descriptive statistics are available in the Online Supplement.

^b^Frequencies and proportions in this table represent weighted sample counts and proportions. Because weighting and rounding can introduce slight discrepancies, totals may not sum exactly to 100% or match the total unweighted sample size.

## Candidate predictors – meta-analytic results

Table 2 shows associations between the 11 childhood candidate predictors and mastery in adulthood; these estimates are from random-effects meta-analyses that combined data from all 22 countries. The effect sizes are displayed in order of strength, with the strongest effects presented first. Some childhood factors, on average across countries in the GFS, were associated with increased mastery in adulthood (e.g., experiencing excellent [RR = 1.08, 95% CI: 1.04, 1.11] or very good health growing up [RR = 1.04, 95% CI: 1.02, 1.06]; regular attendance at religious services at age 12, ranging from at least 1x/week [RR = 1.05, 95% CI: 1.03, 1.08], to 1-3x/month [RR = 1.05, 95% CI: 1.01, 1.10], less than 1x/month [RR = 1.03, 95% CI: 1.01, 1.04]; very/somewhat good relationship with mother [RR = 1.03, 95% CI: 1.01, 1.06], or with father [RR = 1.02, 95% CI: 1.00, 1.04]; living in a family that comfortably met its financial needs [RR = 1.02, 95% CI: 1.00, 1.03]).

**Table 2:** Random effects Meta-Analysis of regression of sense of mastery on childhood predictors.

Variable	Category	RR	95% CI	Estimated proportion of effects by threshold		I^2		Global *p*-value
				< 0.90	> 1.10			
Relationship with mother	(Ref: Very bad/somewhat bad)							0.017*
	Very/somewhat good	1.03	(1.01,1.06)	0.00	0.05	56.9		
Relationship with father	(Ref: Very bad/somewhat bad)							0.009*
	Very/somewhat good	1.02	(1.00,1.03)	0.00	0.00	41.4		
Parent marital status	(Ref: Parents married)							< 0.001**
	No, divorced	0.99	(0.97,1.01)	0.00	0.00	29.5		
	Single, never married	1.01	(0.97,1.05)	0.05	0.14	90.8		
	No, one or both had died	0.98	(0.94,1.01)	0.14	0.05	72.4		
Subjective financial status of family growing up	(Ref: Got by)							< 0.001**
	Lived comfortably	1.03	(1.01,1.05)	0.00	0.05	81.3		
	Found it difficult	0.98	(0.97,0.99)	0.00	0.00	19.4		
	Found it very difficult	0.96	(0.93,0.98)	0.05	0.00	42.6		
Abuse	(Ref: No)							< 0.001**
	Yes	0.96	(0.94,0.98)	0.10	0.00	79.6		
Outsider growing up	(Ref: No)							< 0.001**
	Yes	0.95	(0.93,0.97)	0.09	0.00	63.4		
Self-rated health growing up	(Ref: Good)							< 0.001**
	Excellent	1.08	(1.04,1.11)	0.00	0.18	92.4		
	Very good	1.04	(1.02,1.06)	0.00	0.09	77.1		
	Fair	0.94	(0.92,0.97)	0.27	0.00	67.6		
	Poor	0.93	(0.89,0.97)	0.23	0.00	46.5		
Immigration status	(Ref: Born in this country)							< 0.001**
	No	1.02	(0.99,1.06)	0.00	0.14	78.4		
Age 12 religious service attendance	(Ref: Never)							< 0.001**
	At least 1x/week	1.05	(1.03,1.08)	0.00	0.14	73.9		
	1-3x/month	1.05	(1.01,1.10)	0.00	0.09	90.2		
	Less than 1x/month	1.03	(1.01,1.04)	0.00	0.05	56.7		
Year of birth	(Ref: 1998–2005; age 18–24)							< 0.001**
	1993–1998; age 25–29	1.01	(0.99,1.03)	0.00	0.05	63.8		
	1983–1993; age 30–39	1.03	(1.00,1.07)	0.00	0.23	89.0		
	1973–1983; age 40–49	1.03	(0.98,1.07)	0.14	0.23	92.2		
	1963–1973; age 50–59	1.04	(0.99,1.09)	0.18	0.27	93.6		
	1953–1963; age 60–69	1.04	(0.99,1.10)	0.09	0.36	92.1		
	1943–1953; age 70–79	1.05	(0.99,1.12)	0.14	0.32	90.2		
	1943 or earlier; age 80+	1.07	(0.98,1.16)	0.27	0.50	89.8		
Gender	(Ref: Male)							< 0.001**
	Female	0.98	(0.97,0.99)	0.00	0.00	60.3		
	Other	0.47	(0.13,1.65)	0.56	0.28	99.9		

*p < .05; **p < .004 (Bonferroni corrected threshold).

Other childhood factors were associated, on average across countries in the GFS, with decreased mastery in adulthood (e.g., experienced abuse [RR = 0.96, 95% CI: 0.94, 0.98], felt like an outsider growing up [RR = 0.95, 95% CI: 0.93, 0.97], experienced fair [RR = 0.94, 95% CI: 0.92, 0.97] or poor health growing up [RR = 0.93, 95% CI: 0.89, 0.97], living in a family where it was difficult [RR = 0.98, 95% CI: 0.97, 0.99] or very difficult to meet financial needs [RR = 0.96, 95% CI: 0.93, 0.98], being a female [RR = 0.98, 95% CI: 0.97, 0.99]). However, there was little evidence that, on average, parent marital status or immigration status, were associated with subsequent mastery in adulthood.

### Candidate predictors – do associations vary by country??

When considering country-specific analyses (Supplementary Tables S1A to S22C and Supplementary Figures S1 to S27), some childhood predictors appeared to have a nearly universal positive associations with adult mastery across countries (i.e., living financially comfortably, excellent or very good health growing up, attending religious service at least once a week or 1-3x/month) while others were nearly universally negative (i.e., experiencing poor of fair self-rated health, experiencing a difficult or very difficult financial situation). Still others showed variations. For example, some childhood predictors were mostly positive across countries, including: relationship with mother. Other childhood predictors were mostly negative, including: abuse, feeling like an outsider growing up, being female. Still other childhood predictors showed more mixed effects across countries, including: parents being divorced, single, or one or both parents having died, year of birth/age, immigration status, relationship with father.

There were also interesting patterns and variations among childhood predictors, including year of birth/age (Figures S21-S27). Overall, the meta-analyses showed that mastery generally increased with year of birth year/age. However, heterogeneity in effect estimates also increased with year of birth/age. Specifically, the risk ratios (RR) increased from 1.01 (95% CI: 0.99–1.03) for those aged 25–29 to 1.07 (95% CI: 0.98–1.16) for those aged 80 and older. Additionally, the estimated proportion of effects by threshold for the 25–29 age group indicated that 5% had effects either above 1.10 or below 0.90. However, this combined proportion increased to 23% in the 30–39 age group and further increased to 37% in the 40–49 age group. From ages 50 to 79, the combined proportion remained relatively stable, ranging from 45 to 50%. In the 80 + age group, the combined proportion reached 77%. This indicates not only a general increase in mastery with birth year/age but also a growing variation in effects across different countries as people get older. When considering high-income countries, some displayed increasing mastery with year of birth/age (i.e., Germany, Australia, Japan, United Kingdom, United States, Hong Kong) while other showed a dip right at the oldest ages (i.e., Sweden, Spain, Israel), and one showed a decline in mastery with year of birth/age (i.e., Poland). Other than Poland, four other countries showed a decline in mastery with year of birth/age (i.e., Nigeria, Indonesia, Kenya, Tanzania) and the rest of the countries showed mixed patterns as year of birth/age increased (i.e., Turkey, Mexico, Argentina, Egypt, India, Philippines, South Africa).

Another factor which exhibited considerable heterogeneous effects across countries is childhood health. To facilitate interpretation, effect estimates from the appendix were exponentiated and converted to relative risks (RR). For example, the overall effect size (RR) across all countries for excellent (versus good) childhood health was 1.08 (95% CI: 1.04, 1.11) (Figure S11). Notably, the effect sizes varied between countries, with 7 out of the 9 societies reporting the highest mastery levels being high-income countries, including Japan (RR: 1.49), Sweden (RR: 1.15), the United Kingdom (RR: 1.12), the United States (RR: 1.09), Germany (RR: 1.08), Hong Kong (RR: 1.07), and Australia (RR: 1.06). Conversely, the overall effect size (RR) across all countries for poor (versus good) childhood health was 0.93 (95% CI: 0.90, 0.97) (Figure S14). The effect sizes varied between countries, with 6 out of the 7 societies reporting the lowest mastery levels also being high-income nations, including Hong Kong (RR: 0.53), Japan (RR: 0.79), Sweden (RR: 0.81), the United States (RR: 0.84), Australia (RR: 0.88), and Poland (RR: 0.91). A similar pattern was observed across countries for fair (versus good), where 9 out of the 11 societies reporting the lowest mastery levels were also high-income societies (Figure S13).

### Additional analyses

First, E-values suggested that many of the observed associations were moderately robust to unmeasured confounding (Table 3). For example, for excellent self-rated health, an unmeasured confounder that was associated with both excellent self-rated health and higher mastery by risk ratios of 1.37 each (above and beyond the covariates already adjusted for) could explain away the association, but weaker joint confounder associations could not. Further, to shift the confidence interval to include the null, an unmeasured confounder associated with both excellent self-rated health and higher mastery by risk ratios of 1.25 each could suffice in being able to explain an association, but weaker joint confounder associations could not. Further, the population-weighted meta-analysis that pooled results across countries and factored in sample sizes in each country (Table S23), yielded very similar results to the random-effects meta-analysis. Finally, as a post-hoc sensitivity analysis we analyzed the equivalent results of Table 2 with a different dichotomization point: 1 (always) vs. 0 (often/rarely/never). Results from these analyses (see Table S24) were largely similar to results from Table 2 with some exceptions. For example, the effect estimates for the following factors were larger: being financially comfortable, finding it very financially difficult, excellent health, and attending religious service at least 1x/week.

**Table 3:** Sensitivity of Meta-Analyzed childhood predictors to unmeasured confounding.

Variable	Predictor (level)	E-value for Estimate	E-value for 95% CI
Relationship with mother	(Ref: Very bad/somewhat bad)		
	Very/somewhat good	1.22	1.09
Relationship with father	(Ref: Very bad/somewhat bad)		
	Very/somewhat good	1.15	1.03
Parent marital status	(Ref: Parents married)		
	No, divorced	1.11	1.00
	Single, never married	1.11	1.00
	No, one or both had died	1.18	1.00
Subjective financial status of family growing up	(Ref: Got by)		
	Lived comfortably	1.20	1.13
	Found it difficult	1.15	1.09
	Found it very difficult	1.26	1.16
Abuse	(Ref: No)		
	Yes	1.25	1.14
Outsider growing up	(Ref: No)		
	Yes	1.28	1.21
Self-rated health growing up	(Ref: Good)		
	Excellent	1.37	1.25
	Very good	1.26	1.18
	Fair	1.32	1.22
	Poor	1.36	1.21
Immigration status	(Ref: Born in this country)		
	No	1.17	1.00
Age 12 religious service attendance	(Ref: Never)		
	At least 1x/week	1.29	1.20
	1-3x/month	1.29	1.13
	Less than 1x/month	1.19	1.10
Year of birth	(Ref: 1998–2005; age 18–24)		
	1993–1998; age 25–29	1.12	1.00
	1983–1993; age 30–39	1.21	1.00
	1973–1983; age 40–49	1.19	1.00
	1963–1973; age 50–59	1.25	1.00
	1953–1963; age 60–69	1.25	1.00
	1943–1953; age 70–79	1.28	1.00
	1943 or earlier; age 80+	1.34	1.00
Gender	(Ref: Male)		
	Female	1.18	1.13
	Other	3.69	1.00

## Discussion

In a large, cross-national sample of 22 nations, we examined the associations between 11 childhood candidate predictors and subsequent mastery in adulthood. No single childhood predictor appeared to dominantly influence mastery in adulthood; instead, a combination of several childhood predictors appeared to be at play. Overall, the effect sizes of the various childhood predictors of adult mastery were relatively modest, often corresponding to only a 0.02 or a 0.05 relative risk increase or decrease in mastery. On the other hand, there were a wide range of childhood experiences—such as robust early health, nurturing parental relationships, regular communal or spiritual activities, and financial stability—associated with higher subsequent mastery. Conversely, childhood adversities like experiencing abuse, feeling marginalized, health challenges, and financial difficulties were associated with lower mastery in adulthood. These findings suggest that a combination of several childhood predictors appear to be at play. Our findings converge with prior studies that identified predictors of increased mastery including good health^9,16,22^ and age^54^. Additionally, our observation that feeling like an outsider predicts lower mastery conceptually aligns with similar findings from previous studies. These studies observed that positive social factors, such as higher social participation, social network contact, perceived support from network members are linked with increased mastery^9,16–22^.

Our findings might be understood through a broader application of Bandura’s theory^3^, which posits that a person’s sense of control involves beliefs about personal mastery (i.e., self-efficacy) and perceived constraints (i.e., outcome expectations) and is influenced in part by (a) *firsthand experiences of mastery* and (b) *feedback from interactions with others*, as well as Whitehead’s model of how mastery influences health^10^.

Some of our findings around childhood health (experiencing excellent or very good health growing up) and childhood financial environment (living in a family that comfortably met its financial needs) can be contextualized through the lens of a) *firsthand experiences of mastery*. These are direct experiences that people use to assess their abilities, thereby enhancing their sense of mastery when these experiences are perceived as successful. When children are healthy and financially secure, they have more opportunities to explore novel thoughts and behaviors. This exploration builds skills, psychological resources, behavioral repertoires, and a broad catalogue of mastery experiences that are essential for developing mastery. Conversely, poor childhood health and financial instability limits access to educational, vocational, volunteering, and other opportunities that provide mastery experiences. Additionally, children from families with low socioeconomic status are more likely to face a shortage of resources (e.g., money, knowledge, prestige, power, social connections) necessary for health and well-being, leading to chronic stress^10^. They are also more likely to be exposed to health-damaging environments, like substandard housing conditions, polluted neighborhoods, food desserts, and crime. Together, these factors contribute to higher rates of psychological issues like depression and anxiety, as well as physical health problems like cardiovascular diseases and higher mortality across the lifespan. These adverse health outcomes further undermine the capacity to have mastery experiences throughout life. Additionally, children growing up in lower socioeconomic positions are often socialized to believe they have less control over their destiny, as they face lower expectations from families, teachers, and employers^55,56^. This reinforces lower mastery compared to their more privileged peers.

Some of our findings concerning childhood social factors—such as: (1) a good relationship with mother, (2) a good relationship with father, (3) experiencing abuse, (4) feeling like an outsider, (5) regular attendance at religious services—might be best understood via Bandura’s ideas of (a) *firsthand experiences of mastery* and (b) *feedback from interactions with others*, as well as Lachman’s work on adaptive control processes^57^. Bandura theorized that people can also develop mastery by vicariously observing others performing similar tasks well. This concept can be further contextualized through the idea of “maternal sensitivity” (or “caregiver sensitivity”), which refers to caregivers’ capacity to respond in a predictable, coherent, warm, and accepting manner to a child’s signals, emotions, and behaviors in daily interactions^58,59^. Sensitive parents not only offer warmth, support, and encouragement, they also share the burden of emotion regulation when children are not yet able to do so by themselves^60^. These repeated experiences of attuned responding likely foster children’s confidence, curiosity, and belief in their own capabilities, enabling them to accumulate the firsthand successes that form the basis of mastery. Over time, such sensitivity might scaffold children’s self-regulation skills and bolster their trust that they can influence their environment. Additionally, caregivers with higher “caregiver sensitivity” likely allow children to reflect on these experiences, further enhancing the child’s own mastery. Conversely, reduced or inconsistent caregiver sensitivity (e.g., neglect, abuse, or making a child feel like an outsider) can disrupt early opportunities for exploration and learning, undermine confidence, and contribute to feelings of helplessness. Over time, a persistent lack of attuned support can limit children’s mastery experiences, leaving them less equipped to manage challenges in adulthood. Alongside caregiver sensitivity, constructive family problem-solving has also been highlighted as a potential mechanism for fostering mastery. A longitudinal study observed that adolescents whose families engaged in effective, collaborative resolution of everyday household challenges developed stronger feelings of personal control^61^. These findings align with Bandura’s notion that mastery is cultivated not just by supportive parent-child dynamics but also by observing and participating in confident problem-solving within the broader family system. In addition, Lachman demonstrated that a strong sense of control in mid- and later life can buffer age-related declines through the use of effective strategies and consistent physical activity, suggesting a developmental continuum wherein the quality of early social experiences helps lay the foundation for these adaptive behaviors^57^. If children learn, early on, that they can successfully influence their surroundings they may enter adulthood with a heightened sense of mastery that continues to motivate proactive coping across the lifespan.

We observed heterogeneity in cross-country effects estimates, including year of birth/age (cf. Figures S21-27). Overall, the meta-analyses showed that mastery generally increases with year of birth/age, though heterogeneity in effect estimates also rises. High-income countries such as Germany, Australia, Japan, the United Kingdom, the United States, and Hong Kong displayed increasing mastery with year of birth/age. In contrast, countries like Sweden, Spain, and Israel showed a dip at the oldest ages, while Poland exhibited a decline in mastery with year of birth/age. These trends suggest that stable and high-income economic and political systems foster the ability to achieve higher levels of mastery as people age. However, the observed dips in some countries indicate that even in high-income settings, other factors can impact this trend.

Another childhood factor which exhibited considerable heterogeneous effects across countries was childhood health. In Figure S11, which examined the impact of excellent childhood health on adult mastery, 7 out of the 9 countries reporting the highest mastery levels were high-income countries. Conversely, Figure S14 showed that when childhood health was poor, 6 out of the 7 countries with the lowest mastery levels were high-income nations. Similarly, Figure S13 showed that when childhood health was fair, 9 out of the 11 countries with the lowest mastery levels were also high-income nations. Several factors might explain these observations. High-income countries offer abundant resources, including high-quality: early childhood education, healthcare services, sports and physical activities, and cultural and creative programs—along with relative economic stability and safety. These resources likely benefit children with excellent health, fostering opportunities to explore novel thoughts and behaviors, build skills, and accumulate psychological and social resources. This exploration also likely enhances social skills, confidence, and helps create a broad reservoir of mastery experiences. However, children with poor health may miss out on these benefits due to absenteeism or reduced engagement, limiting their access to critical developmental opportunities. Moreover, high-income societies often emphasize individual achievement and success, which might amplify the effects of childhood health. Children in excellent health can more fully use existing resources and meet high societal standards, strengthening their mastery. Conversely, those with poor health may struggle to harness these resources and face greater challenges and stigmatization, leading to diminished mastery. This diminished mastery might be further exacerbated by the awareness of the abundant resources around them that they are unable to fully engage, due to health limitations.

We also observed that variability in mastery was relatively low in younger age groups, but increased substantially from ages 25–29 to 40–49, as indicated by a growing percentage of countries showing effects above 1.10 or below 0.90. This suggests that as people age, their mastery levels diverge increasingly more across different cultures, potentially due to varying socioeconomic status, healthcare quality, educational opportunities, and cultural values and norms. This variability then plateaued from ages 50 to 79 potentially reflecting a stabilization in life circumstances. However, it rose again in the 80 + age group, highlighting diverse aging processes and the potentially substantial impact of cultural factors on mastery in late adulthood.

This study has several limitations. First, confounding by unmeasured variables is a potential limitation in all observational research. However, we adjusted for a range of potential confounding variables, and we formally evaluated this concern via E-value analyses which suggested that many of the observed associations were moderately robust to unmeasured confounding. Second, the candidate childhood predictors were limited to the factors available in the dataset. Thus, future research with available prospective data should evaluate factors like quality of early childhood education, academic achievement, peer relationships, involvement in extracurricular activities, and early leadership experiences. Third, respondents in different countries might have interpreted the mastery item wording or the response categories differently. However, analyses from cognitive interviews suggest that this was not as severe a problem for mastery as for some of the other items in the Global Flourishing Study^30^. Fourth, the childhood predictors were reported retrospectively, and thus may be subject to recall bias—especially for subjective factors like relationships. Additionally, current mood or adult experiences can influence recall of childhood factors. However, for recall bias to completely explain away the observed associations would require that the effect of adult mastery on biasing retrospective assessments of the childhood predictors would essentially have to be at least as strong as the observed associations themselves (see E-values analyses; also^62^). A few of these (e.g. especially self-rated health) were somewhat larger in magnitude, reducing somewhat the likelihood of this possibility. Fifth, although many of the observed associations in our study were small, they remain meaningful for understanding adult mastery. In psychology, numerous factors jointly shape complex traits, behaviors, and mindsets, and a single variable is rarely expected to account for a large share of variance. Parallel examples from medicine (e.g., aspirin’s r = .03 for preventing heart attacks) and education (e.g., small correlations for various interventions) illustrate that even small effects can generate substantial real-world benefits when aggregated across large populations or over an individual’s lifetime. Sixth, reducing our outcome (i.e., mastery), to a simple “Always/Often” vs. “Rarely/Never” measure, or labeling exposures (e.g., a parental relationship as “Very bad/somewhat bad” vs. “Very/somewhat good’) flattens the variety of real-world experiences that shape a person’s sense of mastery. This shrinking of possibilities may obscure subtler but meaningful patterns, weakening our ability to pinpoint the specific conditions that foster or undermine mastery. We opted for these responses for two primary reasons:^1^ the Global Flourishing Study’s design constraints and pre-registered analytic plan required standardized, easily comparable categories across 22 diverse international samples, and^2^ initial sensitivity analyses showed that our main findings remained robust even under alternative cut points. For example, when considering our outcome, we initially chose this cutoff because it reflects a conceptual distinction where individuals who consistently feel capable (“Always/Often”) are separated from those who have a pronounced deficit in perceived capability (“Rarely/Never”). To evaluate if the main findings hinge on the exact cutoff point we chose, we conducted a post-hoc sensitivity analysis, where mastery was analyzed with a different dichotomization point: 1 (always) vs. 0 (often/rarely/never). Results indicated that some predictors (e.g., robust childhood health, comfortable finances, frequent religious attendance) showed larger effect sizes under the stricter definition, whereas weaker associations diminished further, making our original (Always/Often vs. Rarely/Never) cutoff relatively more conservative by comparison. Despite these shifts, the overall pattern remained consistent, suggesting that focusing on those who feel unfailingly capable accentuates certain childhood pathways but does not materially alter our central conclusions. Overall, our analyses indicated that this dichotomization, supported by both conceptual and methodological reasons, is sufficiently robust and captures meaningful differences in mastery. Seventh, we decided not to model the four-category mastery item (“Never, Rarely, Often, Always”) as a continuous outcome for two reasons. First, the item offers fewer than five ordered response options. Simulation work shows that when ordinal variables have only two to four categories, treating them as continuous with standard regression methods that assume equal spacing produces downward-biased coefficients and overly narrow standard errors^63^. However, categorical approaches remain unbiased in such circumstances. Second, the response distributions are heavily skewed, and the degree of skew differs across the 22 countries. Under such asymmetry, interval models become still more biased, whereas multilevel logistic models that keep the variable categorical remain accurate.

This study also had notable strengths. It was among the first studies to evaluate childhood predictors of mastery, and also among the first studies to evaluate this question cross-nationally. Further, we used a large, diverse, and nationally representative samples from 22 countries. We also were able to use well-thought out questionnaires that were explicitly designed for the GFS, a panel study of well-being (with four additional waves of data to be collected annually from 2024 to 2027).

Our findings highlight the potentially fundamental role of childhood experiences—such as robust early health, nurturing parental relationships, regular communal or spiritual activities, and financial stability—in enhancing an adult’s sense of mastery. Conversely, adversities like experiencing abuse, feeling marginalized, health challenges, and financial difficulties were associated with lower mastery in adulthood. Further, our exploration into the heterogeneity of early life conditions and their impact on adult mastery across 22 countries highlights the nuanced roles that early health and year of birth/age play in shaping adult mastery across nations. With further research, these findings could inform the development of globally adaptable, yet locally nuanced, programs and policies that target childhood factors in order to enhance mastery in adulthood^64,65^.

## Electronic supplementary material

Below is the link to the electronic supplementary material.

Supplementary Material 1

## Data Availability

Data are available for download at https://www.cos.io/gfs. Code Availability: All analyses were pre-registered with COS prior to data access (https://osf.io/jc2mr); all code to reproduce analyses are openly available in the online OSF repository.
